# A TRPA1-mediated substance P release assay as an *in vitro* screening tool for airway irritant potential

**DOI:** 10.3389/ftox.2026.1870419

**Published:** 2026-08-24

**Authors:** R. Bedford, R. Medhane, J. Lawrance, M. Hollings

**Affiliations:** Labcorp, Harrogate, United Kingdom

**Keywords:** adverse outcome pathways, airway irritation, new approach methodology, respiratory, TRPA1

## Abstract

Environmental exposure to airway irritants can elicit sensory irritation and contribute to various disease outcomes. Although *in vitro* assays have been developed to measure skin and eye irritation, evaluation of respiratory sensory irritation relies predominately on *in vivo* testing using the RD_50_ assay, or Alarie test. Although the RD_50_ assay aligns well with human responses, various disadvantages, such as low throughput and interspecies differences in receptors responsible for detecting noxious stimuli, highlights a few of the many requirements for alternative, or complementary *in vitro* approaches. Recently, increased emphasis has been placed on methods that predict human responses at the mechanistic level. In this study, we have developed a human relevant *in vitro* model for airway irritant detection based around molecular events of airway irritation, including transient receptor potential channel activation and substance P release. Using alveolar- and bronchial-derived cell lines, we demonstrate endogenous TRPA1-driven Substance P release following exposure to prototypical irritants. Calcium response was confirmed by flow cytometry following exposure to irritants, with substance P release demonstrated at both the gene and protein levels by qPCR, immunocytochemistry and ELISA. Furthermore, our model demonstrates some concordance with *in vivo* RD_50_ data.

## 1 Introduction

Environmental exposure to airway irritants can trigger a range of acute respiratory symptoms, including pain, cough, dyspnea, and bronchospasm. Chronic or repeat exposure can lead to longer term conditions such as airway hyper-responsiveness, asthma, and chronic obstructive pulmonary disorder (COPD). These disorders contribute to reduced economic productivity and increased public health costs. The use of volatile organic chemicals (VOCs) in consumer products including those found in solvents, vapours and gases continues to increase. Furthermore, many of these VOC’s are released into the environment and remain poorly characterised with respect to their potential to cause airway irritation (Wallbanks et al., 2024; Jung et al., 2025; Dalton, 2003).

The human respiratory tract is composed of various cell types, such as epithelial cells, that express transient receptor potential (TRP) ion channels. Activation of these TRP ion channels has been described as a front-line mechanism for detecting environmental irritants (Gerhold and Bautista, 2008). TRPA1 and TRPV1 are two key ion channel receptors present in the airways that mediate irritation as part of a physiological response to make the subject aware of the presence of chemicals and initiate several defensive biological responses. The activation of TRPA1 and TRPV1 has been well characterised as a gatekeeper of inflammation and is an important process in regulating the airway response following exposure to VOCs (Bautista et al., 2013; Müller et al., 2022; Bessac and Jordt, 2008).

Chemical irritants can be identified based on physical and chemical properties. Traditional assays used to evaluate irritancy include the *in vitro* skin and eye corrosion/irritation assays (Lee et al., 2017; OECD, 2015; OECD, 2016). However, respiratory irritation is more complex to measure *in vitro* and currently used assays involve animal models. In these models, corrosive effects are identified through histopathological endpoints, such as cell necrosis and inflammatory cell infiltration. Whilst these observations are predictive for corrosive effects, in general, they are not predictive of sensory irritation (Bos et al., 2002). Determination of sensory irritation is limited to the *in vivo* sensory irritation test that measures the 50% reduction in respiration rate or RD_50_, also known as the Alarie test (Alarie, 1981). Although this assay demonstrates correlation with irritancy in humans for certain compounds, limitations include inability to predict human mechanistic responses and lack of cost-effectiveness for high-throughput screening of potential irritants. Furthermore, limitations with currently used risk assessment strategies appear to be reflected in epidemiological data with healthcare and cleaning occupations demonstrating increased occurrences of irritant-induced asthma associated with irritant exposure levels below established occupation exposure limits (OELs) (Baur et al., 2012; Bos et al., 2002). The use of mode-of-action testing has been suggested for setting air quality guidelines. Furthermore, the ability to develop systematic approaches using *in vitro* techniques aligns with the changing landscape of risk assessment testing, with mechanistic testing, such as the use of adverse outcome pathways (AOP), becoming more important (Wallbanks et al., 2024; Martinez and Eling, 2019; Kim and Choi, 2026).

In this study, we developed an *in vitro* airway irritation model based on TRPA1 and TRPV1 activation, quantified using flow cytometry. We demonstrate the expression of TRPA1 and TRPV1 in the human alveolar cell line, A549, following which activation of the channels is confirmed using a combination of channel-specific agonists and inhibitors. Subsequently, we confirmed the effects of several known airway irritants on TRPA1 and TRPV1 activation using the developed model. Using previously described molecular pathways, we investigated the link between TRP channel activation and substance P expression–a downstream signalling mechanism known to drive the airway irritation response in humans (Martinez and Eling, 2019). The presence of TRPA1-gated calcium responses and substance P release following exposure to a panel of known airway irritants highlighted the potential use of our model as a novel *in vitro* tool for screening compounds for their irritant potential in the respiratory tract. Finally, we confirmed the presence of similar biomarkers in the human upper airway model, EpiAirway™.

In conclusion, we describe the development of promising human alveolar- and bronchial-derived *in vitro* models that could be used alongside current approaches, such as the Alarie test, during the assessment of compounds in their ability to induce respiratory irritation. Such an approach could help to improve cost-efficiency and reduce animal use during compound risk assessment.

## 2 Materials and methods

### 2.1 Chemicals tested in the study

All chemicals tested in the study were purchased from Sigma-Aldrich (Merck KGaA, Darmstadt, Germany). The chemicals tested include; Allyl Isothiocyanate (AITC) (CAS No. 57–06–7), Capsaicin (CAS No. 404–86–4), acetone (CAS. No. 67–64–1), Nicotine (CAS. No. 54–11–5), p-Xylene (CAS. No. 106–42–3), Methanol (CAS. No. 67–56–1), Toluene (CAS. No. 108–88–3), Heptanal (CAS. No. 111–71–7), 2-Ethoxy Ethyl Acetate (CAS. No. 111–15–9), formaldehyde (CAS. No. 50–00–0), Isopropanol (CAS. No. 67–63–0), acetic acid (CAS. No. 64–19–7), HC-030031 (CAS. No. 349085–38–7) and Capsazepine (CPZ) (CAS. No. 138977–28–3). These chemicals were tested in this study to provide a range of responses based on responses observed in animal models (Schaper, 1993).

### 2.2 Cell culture

A549 cells were purchased from Culture Collections (Porton Down, Wiltshire) and cultured in Roswell Park Memorial Institute (RPMI) 1,640 medium containing 2 mM glutamine (Cat. No. 11875093, Thermo Fisher Scientific, Waltham, MA, United States) supplemented with 10% fetal bovine serum (FBS) (Cat. No. F9665, Sigma-Aldrich, Merck KGaA, Darmstadt, Germany) and 1% penicillin and streptomycin (Cat. No. P4333, Sigma-Aldrich, Merck KGaA, Darmstadt, Germany). Cells were provided by the vendor at passage 91 and used in experiments at passage 93 with routine *mycoplasma* testing performed by *Mycoplasma* Experience (*Mycoplasma* Experience Limited, Surrey, United Kingdom). One day before treatment, A549 cells were seeded at a cell density of 6 × 10^4^ cells per well in 24-well plates. A549 cells were grown in 1.0 mL medium until treatment commenced. For viability measurement using WST-8, cells were treated with 1.0 mL of the appropriate compound at specified concentrations for a 24 h duration. Compound treatments for the calcium response assay are described in subsequent sections.

EpiAirway™ tissues were purchased in 12-well Transwell inserts from MatTek (Ashland, MA, United States) and cultured in proprietary EpiAirway™ Maintenance Medium (AIR-100-MM; MatTek) according to the manufacturer’s protocol. EpiAirway™ tissues were maintained at the air liquid interface (ALI) with 1 mL medium in the basal compartment with medium replaced every 24 h and experiments performed 7 days following tissue culture set-up. EpiAirway™ tissues were of bronchial origin from a healthy donor.

### 2.3 Viability measurements by WST-8 assay

Following the 24 h treatment duration, treatment medium was removed from cells and the contacted surface rinsed with calcium and magnesium-free PBS (CMF-PBS) (Cat. No. 10010023, Thermo Fisher Scientific, Waltham, MA, United States). Viability was measured using the WST-8 assay. Briefly, 0.4 mL of WST-8 solution (Cat. No. 96992, Merck KGaA, Darmstadt, Germany), diluted 1 in 10 using appropriate medium, was applied to A549 cells, or basolateral surface of EpiAirway™ tissues, for 1 h. Subsequently, 0.1 mL aliquots were plated in triplicate and absorbance measured at 450 nm. To determine IC_20_ and IC_50_ values, viability was expressed as relative viability in comparison to the vehicle control and cell viability was interpolated to assign the concentrations required to reach 80% and 50% viability respectively. Cells were rinsed with CMF-PBS and stored at < -50 °C for future qPCR analysis.

### 2.4 RNA analysis

Total RNA was isolated from A549 cells and EpiAirway™ tissues following treatment using the RNAqueous™ Total RNA Isolation Kit (Cat. No. AM 1912, Thermo Fisher Scientific, Waltham, MA, United SA) according to the manufacturer’s instructions. A single EpiAirway™ tissue was used for each treatment condition per experiment, in three independent experiments, resulting in three tissues per treatment condition in total (n = 3). A549 measurements were performed in duplicate, per experiment, resulting in six individual replicates analysed in total. RNA concentration and purity was measured at 260 nm/280 nm by spectrophotometry. RNA concentrations were normalized to 100 ng of RNA per reaction and converted to complementary DNA (cDNA) using QuantiTect® Reverse Transcription Kit (Cat. No. 205311, Qiagen, Hilden, Germany). TaqMan® Gene Expression primer/probes (Thermo Fisher Scientific, Waltham, MA, United States) were purchased for the following genes: *GAPDH*, *TRPA1*, *TRPV1*, *TAC1*. Quantitative PCR (qPCR) was performed using 1 ng cDNA per reaction with each primer/probe added to separate wells with the PCR performed as single plex reactions. Subsequently, qPCR was performed using a QuantStudio™ 5 Real-Time PCR System (Thermo Fisher Scientific, Waltham, MA, United States) and data was analysed by relative quantification using the ΔΔCt method and quantification normalized to the endogenous control, *GAPDH*. Throughout the study, GAPDH Ct values measured 17.898 ± 0.561 SD. Normalised values were subsequently compared to the normalized vehicle control values to calculate the RQ, or fold-change, values. All data calculations were performed using QuantStudio™ 5 Design and Analysis Software version 1.5.2.

### 2.5 Calcium influx using flow cytometry

Intracellular calcium influx was assessed in A549 and EpiAirway™ cells using the calcium-sensitive fluorescent dye Fluo-4, AM. A549 or EpiAirway™ cells were incubated with 1 μM Fluo-4, AM (Cat. No. F14201, Invitrogen, Thermo Fisher Scientific, Waltham, MA, United States) for 1 hour at 37 °C ± 1 °C in an atmosphere of 5% ± 1% (v/v) CO_2_ in air. Fluo-4, AM was reconstituted in DMSO to a stock concentration of 1 mM and diluted in appropriate medium to achieve the final working concentration (1 μM).

Cells were washed three times with Hank’s Buffered Saline Solution (HBSS) containing Ca^2+^ and Mg^2+^ and incubated in HBSS containing Ca^2+^ and Mg^2+^ for 30 min at room temperature protected from light to allow complete de-esterification of intracellular AM esters. Cells were trypsinised using 0.25% Trypsin-EDTA until detached and cells were then centrifuged and resuspended in HBSS (containing Ca^2+^/Mg^2+^). HC-030031 or capsazepine (CPZ) were added to cells for TRPA1 or TRPV1 inhibition, respectively, for 30 min on ice. In addition, cells were supplemented with 1 μg/mL propidium iodide as a cytotoxicity marker. Cells remained on ice until agonist addition and measurement of intracellular calcium movement by flow cytometry.

Flow cytometry measurements were performed using a BD FACSCanto™ II (3 lasers, 4-2–2 configuration, BD Biosciences, Franklin Lakes, NJ, United States). During measurement of calcium response, 0.1 mL of sample supplemented with 0.1 mL HBSS (containing Ca^2+^/Mg^2+^) (baseline state) was recorded for 3,000 events prior to immediate measurement of an additional 3,000 events following addition of agonist compound (activation state). Activation by agonist was measured immediately on addition until 3,000 events were acquired, with the mean FITC measurement used to assess level of calcium influx, and binding to Fluo-4, AM. The mean FITC measurement from the activated state was subtracted from the mean FITC measurement from the baseline state. Normalisation to the maximum response induced by 1 μg/mL ionomycin and the vehicle control only response was subsequently performed to calculate a percentage maximum calcium response. In addition, to control for potential fluorescence drift during the assay, for example, from measurement of the first concentration of compound to measurement of the final concentration of compound, baseline fluorescence measurements were taken before each concentration of compound.

### 2.6 Detection of hTRPA1 and hTRPV1 using flow cytometry

A549 cells were cultured as previously described following which cells were trypsinised, pelleted and fixed using 3% paraformaldehyde for 1 h at room temperature. Cell pellet was washed and cells permeabilised for 1 h using 1% (v/v) Triton X-100 in PBS. Fixed and permeabilised cells were incubated with anti-TRPA1 (Cat. No. PA1-46159, Thermo Fisher Scientific, Waltham, MA, United States) and anti-TRPV1 (Cat. No. PA1-748, Thermo Fisher Scientific, Waltham, MA, United States) specific antibodies overnight at 4 °C. Cells were washed and incubated overnight using Alexa Fluor™ 488 conjugated anti-Rabbit secondary antibodies (Cat. No. A-11008, Thermo Fisher Scientific, Waltham, MA, United States). Detection of bound fluorophore-tagged secondary antibodies was performed using a BD FACSCanto™ II (3 lasers, 4-2–2 configuration, BD Biosciences, Franklin Lakes, NJ, United States). In addition, cells incubated with buffer only and secondary antibody only were analysed for to confirm background fluorescence levels.

### 2.7 Detection of substance P in A549 cells using immunocytochemistry

A549 cells that had been treated for a 24-h duration were washed and subsequently fixed using 3% paraformaldehyde for 1 h at room temperature and permeabilised for a further 1 h using 1% (v/v) Triton X-100 in PBS. Fixed and permeabilised cells were incubated with anti-substance P antibody (Cat. No. Ab14184, Abcam, Cambridge, United Kingdom) overnight at 4 °C. Cells were washed and incubated with Alexa Fluor™ 647-conjugated anti-mouse antibody (Cat. No. Ab150115, Abcam, Cambridge, United Kingdom) and Hoechst 33,258 solution (Cat. No. 94403, Sigma-Aldrich, Merck KGaA, Darmstadt, Germany) overnight at 4 °C. Detection of fluorophore-tagged secondary antibodies was performed using an EVOS™ M3000 Imaging System (Cat. No. AMF3000, Thermo Fisher Scientific, Waltham, MA, United States). ImageJ v1.47 was subsequently used to calculate ratios between the blue (nuclei-staining) and red (substance P-staining) channels to determine overall substance P levels.

### 2.8 Detection of secreted substance P in EpiAirway™ by ELISA

Following treatment with compound for a 24 h duration, medium was recovered from EpiAirway™ tissues and used to measure secreted substance P by ELISA (Cat. No. EEL013, Thermo Fisher Scientific, Waltham, MA, United States) according to manufacturer’s instructions. Briefly, 50 μL of sample was added to the ELISA plate for 45 min at 37 °C and then washed three times. 100 μL HRP-Conjugate was added to each well for 30 min at 37 °C and washed five times. Subsequently, 90 μL Substrate Reagent was added to each well for 15 min at 37 °C followed by addition of 50 μL Stop Solution. The plate was measured at 450 nm using a spectrophotometer and data analysed by subtracting the medium only values from each sample value. Fold-change compared to vehicle control was calculated. In addition, a standard curve was used to interpolate substance P concentration in each sample (pg/mL) using a four-parameter logistic curve.

### 2.9 Statistical analysis

Data are presented as the mean ± SEM (unless otherwise stated) of three independent experiments. All statistical analyses were performed using GraphPad Prism ®Software 10.4.2 (GraphPad Software Inc., La Jolla, CA, United States). All data was tested for normality using the Shapiro-Wilk normality test. Subsequently, comparisons between groups were performed by one-way analysis of variance (ANOVA). Values of *p* < 0.05 were considered to be statistically significant. Dose-response data were fitted using a four-parameter logistic (4 PL) model to accommodate flexible estimation of minimum and maximum responses and provide an appropriate sigmoidal fit.

## 3 Results

### 3.1 Assessment of TRPA1 and TRPV1 expression in A549 cells

**FIGURE 1:**
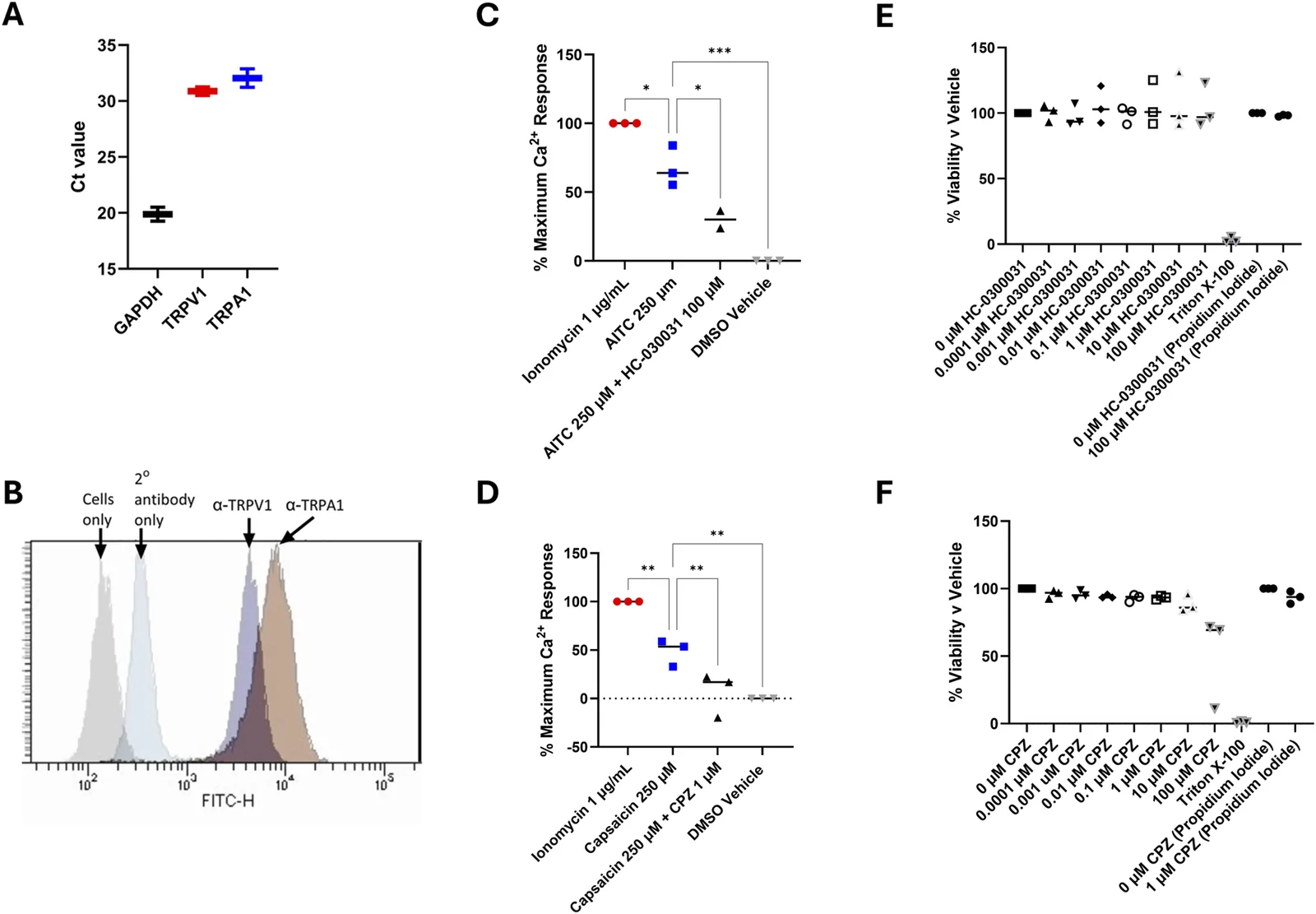
Confirmation of TRPA1 and TRPV1 expression and function in A549 cells. Expression of TRPA1 and TRPV1 mRNA and protein in A549 cells confirmed by qPCR **(A)** and flow cytometry **(B)**, respectively. Confirmation of functional activation of TRPA1 **(C)** and TRPV1 **(D)** in A549 cells was performed using specific agonists and antagonists. TRPA1 activity was confirmed using the agonist AITC and antagonist HC-030031, while TRPV1 activity was confirmed using the agonist capsaicin and antagonist, capsazepine (CPZ). In addition, A549 viability was measured by WST-8 following treatment with HC-030031 **(E)** and CPZ **(F)** at a range of concentrations to confirm viability at concentrations used in activation assays. Viability at the selected concentration of antagonist to be used during subsequent assays was also measured using propidium iodide **(E,F)**. Mean ± SEM (n = 3). One-way ANOVA with multiple comparisons. *p < 0.05, **p < 0.01, ***p < 0.001.

To confirm that A549 cells were an appropriate model for testing compound risk for airway sensory irritation induction, the presence of TRPA1 and TRPV1 ion channels was initially confirmed by qPCR (Figure 1A). Although expression was relatively low in comparison to the endogenous gene control, *GAPDH*, Ct values of 30.872 and 32.047 for TRPV1 and TRPA1, respectively, confirmed their presence. Despite this, gene expression does not necessarily confirm functional protein presence.

To confirm the presence of TRPA1 and TRPV1 protein in A549 cells, cells were labelled with anti-TRPA1 and anti-TRPV1 antibodies respectively, with detection performed by flow cytometry (Figure 1B). Increased fluorescence intensity of anti-TRPA1 and anti-TRPV1-labelled cells compared to cells only demonstrated increased expression of both channels. Furthermore, secondary-antibody only labelled cells demonstrated lower fluorescence intensity than the primary antibody-labelled cells, demonstrating that the fluorescence intensity was not a result of the secondary antibody binding to non-specific targets in A549 cells. It should be noted however, that due to the primary antibody epitopes being present on the C-terminus of the TRPA1 and TRPV1 channels, the cells had to be permeabilised to enable antibody access to their targets. Consequently, the increased signal does not necessarily confirm channel presence at the cell membrane, or channel functionality. Furthermore, despite the higher fluorescent signals observed following use of TRPA1 and TRPV1-specific antibodies, the high concentration of Triton X-100 (1%) used to permeabilise the fixed cells may have resulted in potential extraction of the ion channels from the membrane, further supporting the possibility that antibodies were binding to intracellular targets.

To confirm function of the expressed ion channels, treatment with TRPA1 and TRPV1-specific agonists and antagonists was performed and calcium mobilisation confirmed using the calcium probe, Fluo-4, AM, with measurement performed by flow cytometry using previously described methods, with minor adjustments (Bajnok et al., 2023). During calcium measurement, the calcium ionophore, ionomycin, was used to determine a maximum calcium response (100%) and the vehicle control, DMSO, used to determine the minimum calcium response (0%). The TRPA1 channel agonist, allyl isothiocyanate (AITC) was added to cells at a final concentration of 250 μM with a mean calcium response measured as 67.72% (±14.66%) activation (compared to the 100% ionomycin response). In contrast, treatment with AITC after a 30-min treatment with the TRPA1 antagonist, HC-030031, at a concentration of 100 μM resulted in a mean calcium response of 30.10% (±8.92%), with responses in the absence and presence of antagonist demonstrating a significant difference (*p* < 0.05) (Figure 1C). To confirm TRPV1 channel function, the TRPV1 channel agonist, capsaicin, was added to cells at a final concentration of 250 μM with a mean calcium response measured as 48.47% (±13.71%) activation. In contrast, treatment with capsaicin after a 30-min treatment with the TRPV1 antagonist, capsazepine (CPZ), at a concentration of 1 μM, resulted in a mean calcium response of 6.49% (±22.90%), with responses in the absence and presence of antagonist demonstrating a significant difference (*p* < 0.01) (Figure 1D). To confirm that the reduced responses observed in the presence of antagonist were not driven by toxicity, A549 viability was measured following treatment with a range of concentrations of HC-030031 (Figure 1E) and CPZ (Figure 1F), with no, or limited, loss in viability observed at the selected concentrations of HC-030031 (100 μM) or CPZ (1 μM), suggesting that loss of channel activation was a direct result of inhibition, rather than an artefact of toxicity. Viability following treatment at the selected concentrations of HC-030031 (98.1% v vehicle control) and CPZ (93.5% v vehicle control) was also confirmed during the calcium response assay by measuring propidium iodide uptake (Figures 1E,F), again, with minimal decrease in viability measured.

### 3.2 TRP channel activation assessed by a panel of known airway irritant compounds

**FIGURE 2:**
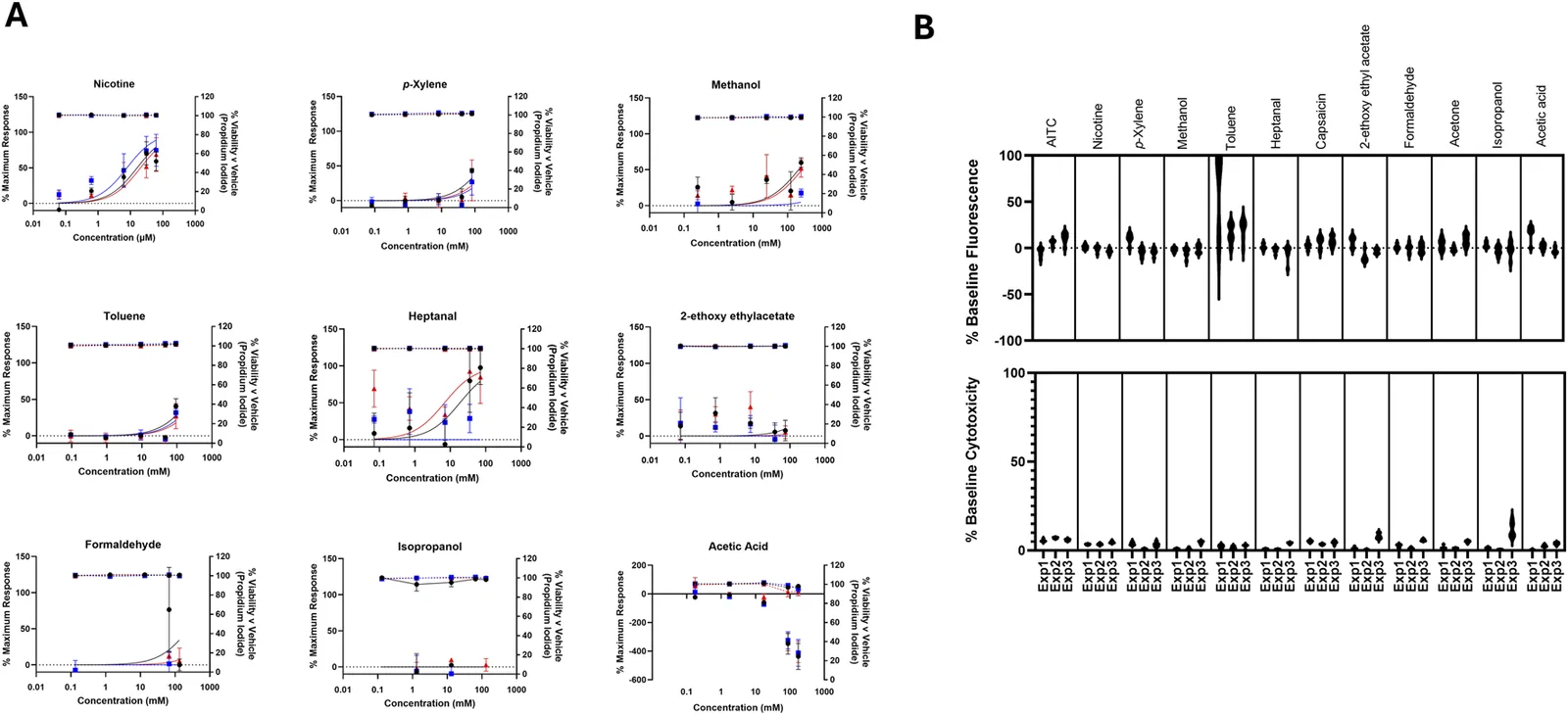
Use of flow cytometry-based calcium-flux assay for testing airway irritants. The developed flow cytometry-based calcium-flux assay was used to test additional compounds identified as airway irritants or non-irritants in animal studies, with non-inhibited (black), TRPA1 inhibited (blue) and TRPV1 inhibited (red) responses, as a percentage of the maximum ionomycin response, shown (solid line). Viability following application of compound at each concentration was also measured using propidium iodide (dashed line) **(A)**. In addition, assay fluorescence baseline and cell toxicity, using propidium iodide, were measured throughout the assay procedure **(B)**. Mean ± SEM (n = 3).

Following successful use of the flow cytometry-based TRP channel activation assay using established agonists and antagonists, we next assessed its suitability for screening airway irritant potential by testing a panel of compounds with known *in vivo* irritancy profiles. A range of calcium responses were observed across the panel (Figure 2A) with statistical significance outlined (Table 1). For example, nicotine induced a marked increase in intracellular calcium that was unaffected by TRPA1 or TRPV1 inhibition, an expected response considering the role of nicotinic acetylcholine receptors (nAChRs) in nicotine binding and the high expression levels observed in A549 cells (Sun et al., 2017; Mucchietto et al., 2018). Interestingly, *p*-xylene and toluene have previously demonstrated TRPA1 and TRPV1mediated responses in mouse models (Sándor et al., 2009; Norões et al., 2019). Although a significant difference was not observed when comparing non-inhibited and TRPA1/TRPV1-inhibited responses in our model, non-inhibited responses did demonstrate a slightly greater response compared to TRPA1 and TRPV1 inhibited responses. Overall, non-inhibited percentage maximum calcium response to *p*-Xylene treatment was 43% (*p* < 0.01 v vehicle control) while responses following TRPA1 inhibition and TRPV1 inhibition were 27% and 29%, respectively, neither of which were significantly different compared to vehicle control response. Following toluene treatment, non-inhibited percentage maximum calcium response was 40% (*p* < 0.0001) compared to TRPA1 (*p* < 0.001) and TRPV1 (*p* < 0.01) inhibited responses of 32% and 27%, respectively. In contrast, 2-ethoxyethyl acetate and isopropanol did not induce statistically significant calcium responses. It should be noted however, that formaldehyde responses were inconsistent. Despite this, in comparison with previous studies, a minor response was observed (*p* < 0.05) that was not observed following TRPA1-and TRPV1 inhibition (Macpherson et al., 2007; Song et al., 2017). Notably, acetic acid produced a pronounced, concentration-dependent decrease in calcium signal, highlighting the challenges of measuring calcium dynamics for certain compounds. For example, even low concentrations of acetic acid may result in disrupted membrane integrity and toxicity, even during short-term application, thereby promoting leakage of calcium sensitive dyes from cells. Indeed, propidium iodide data suggests greater toxicity induction following acetic acid application, in comparison to other compounds. Alternatively, the reduced pH following acetic acid addition may result in reduced fluorescence intensity of calcium probes such as Fluo-4, AM via a quenching mechanism (Maruta and Yamashita, 2020; Baker et al., 1994; Rohrbach et al., 2005).

**TABLE 1:** Statistical significance using one-way ANOVA with multiple comparisons observed for calcium responses following treatment with airway irritant compounds as presented in Figure 2A.

Compound	Non-inhibited significance (v untreated)	TRPA1 inhibited significance (v untreated)	TRPV1 inhibited significance (v untreated)
Nicotine	1.5 μM: *p* < 0.001 1 μM: *p* < 0.01	0.1 μM: *p* < 0.05 0.5 μM: *p* < 0.001 1 μM: *p* < 0.001	0.1 μM: *p* < 0.05 0.5 μM: *p* < 0.05 1 μM: *p* < 0.01
p-Xylene	1 mM: *p* < 0.01	NS	NS
Methanol	1 mM: *p* < 0.01	NS	1 mM: *p* < 0.01
Toluene	1 mM: *p* < 0.0001	1 mM: *p* < 0.001	1 mM: *p* < 0.01
Heptanal	0.5 mM: *p* < 0.05 1 mM: *p* < 0.05	NS	0.5 mM: *p* < 0.05 1 mM: *p* < 0.05
2-Ethoxy ethylacetate	NS	NS	NS
Formaldehyde	0.5 mM: *p* < 0.05	NS	NS
Isopropanol	NS	0.5 mM: *p* < 0.05	NS
Acetic acid (decreased response)	0.5 mM: *p* < 0.0001 1 mM: *p* < 0.0001	0.5 mM: *p* < 0.001 1 mM: *p* < 0.0001	0.5 mM: *p* < 0.001 1 mM: *p* < 0.0001

To further evaluate assay performance, we monitored baseline fluorescence stability and cell viability throughout the experimental window (Figure 2B). As the assay relies on repeated baseline measurements between compound additions, minimal variability in these readings, as well as sustained cell health, is essential. Substantial declines in baseline fluorescence, for instance, could indicate dye leakage and compromise interpretation of compound-induced responses. Overall, baseline fluorescence remained stable across experiments for most compounds, although measurements during toluene testing were more variable. Although there is the possibility that this is a result of the high volatility of toluene, or autofluorescence of residual toluene remaining in the flow cytometer during baseline measurements, this is unlikely as stable baseline fluorescence was observed for the similar aromatic hydrocarbon compound, *p*-xylene. Nevertheless, further investigations would be required to further understand the fluorescent drift observed during toluene testing. Cell viability remained high throughout for all compounds tested, with toxicity consistently below 10%, as measured by propidium iodide uptake, supporting the robustness of the assay conditions.

### 3.3 Activation of TRPA1 in A549 cells promotes substance P signalling

Paracrine communication between neuronal and epithelial cells has been identified as a key driver of airway sensory irritation and subsequent inflammatory responses. A key peptide in this signaling cascade is substance P, expressed from the preprotachykinin-1 gene, *Tac1*. Although previous studies have demonstrated oxidative stress and inflammatory induction following substance P expression in neuronal cells, or in A549 cells following treatment with exogenous substance P, we sought to determine the ability of A549 cells to express endogenous substance P following activation of TRPA1 and TRPV1 (Xu et al., 2018; Springer et al., 2005; Nguyen et al., 2023).

**FIGURE 3:**
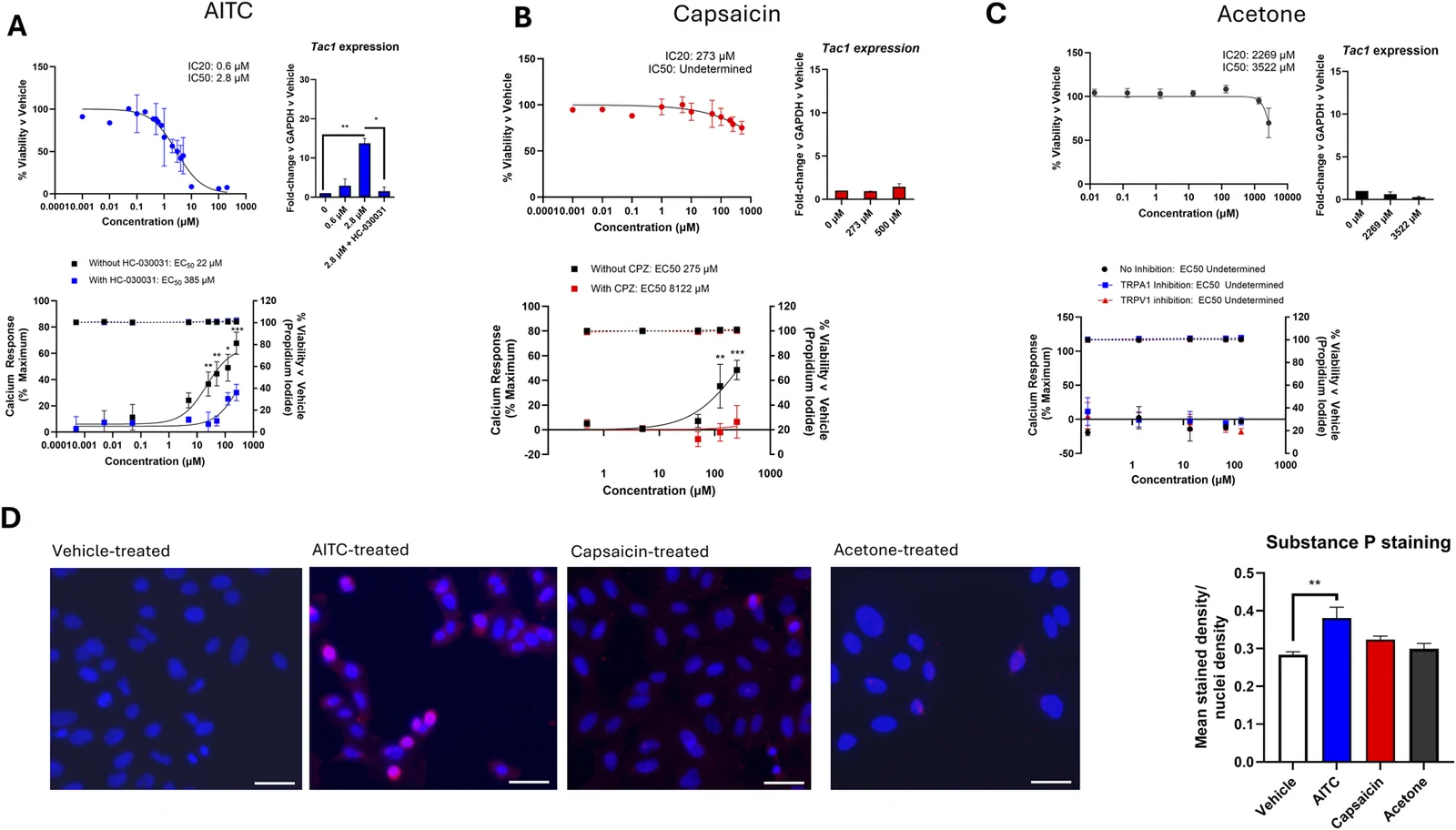
Confirmation of biomarker presence associated with airway sensory irritation. Activation of TRPA1 in A549 cells using the known agonist, AITC, was measured using Fluo-4,AM, with measurements performed on immediate application of AITC. Calcium responses were also measured following treatment with 100 μM HC-030031 for 30 min prior to agonist application. Viability following agonist application was measured using propidium iodide during the calcium response assay. Furthermore, treatment with AITC for 24 h induced a concentration-related decrease in cell viability, as measured by WST-8, with a calculated IC_50_ of 2.8 μM–a concentration that induced a significantly increased expression of *Tac1* in A549 cells. This increased expression was inhibited by the presence of 1 μM HC-030031 for the 24 h treatment duration **(A)**. These responses were not observed with capsaicin **(B)** or acetone **(C)**. Confirmation of increased substance P expression by immunocytochemistry following a 24 h AITC treatment duration. Scale-bar: 100 μm, ×20 magnification **(D)**. Mean ± SEM (n = 3). One-way ANOVA with multiple comparisons. *p < 0.05, **p < 0.01, ***p < 0.001.

Initially, A549 cells were treated with AITC at a range of concentrations for a 24 h duration and viability measured, with IC_20_ and IC_50_ values of 0.6 μM and 2.8 μM determined, respectively. Subsequently, using the flow cytometric-based calcium mobilization assay described in Figures 1, 2, the ability of AITC to evoke a concentration-related response was investigated in the absence (EC_50_ 22 μM) and presence (EC_50_ 385 μM) of 100 μM HC-030031, with a significant difference observed. Furthermore, a significant increase in endogenously expressed *Tac1* was confirmed by qPCR following treatment with 2.8 μM AITC for 24 h (*p* < 0.01), an increase that was significantly diminished in the presence of 1 μM HC-030031 (*p* < 0.05) (Figure 3A). It should be noted that although only a small increase in *Tac1* expression (not statistically significant) was observed following AITC treatment at 0.6 μM, this may have been a result of variability in viability, with viability being as high as 90% during the *Tac1* expression experiments presented in Figure 3.

Following treatment with capsaicin, although decreased cell viability and increased calcium mobilization was observed, increased *Tac1* expression was not observed at the capsaicin concentrations tested (Figure 3B). However, it should be noted that higher concentrations of capsaicin may have induced higher levels of *Tac1* expression. Alternatively, the lack of *Tac1* expression following capsaicin-induced TRPV1 activation, may be explained by expression of the ion channel receptor on an intracellular organelle, rather than at the plasma membrane. Both possibilities should be further explored in future studies. Treatment with acetone, a compound that has demonstrated low respiratory irritation when tested using *in vivo* models, demonstrated negligible toxicity, calcium response or *Tac1* expression (Figure 3C). To confirm that increased *Tac1* expression following treatment with 2.8 μM AITC translated to enhanced substance P protein expression, immunocytochemistry was performed and substance P-specific staining intensity normalized to nuclei intensity (Figure 3D). Results demonstrated a significant increase in substance P staining following treatment with AITC compared to vehicle treatment (*p* < 0.01). A statistically significant increase was not observed following treatment with capsaicin (500 μM) or acetone (3,522 μM). The endogenous gene control, *GAPDH*, response remained consistent during compound treatments, with an average Ct value of 17.571 ± 0.284 SD observed.

### 3.4 Viability, calcium responses and *Tac1* expression following treatment with known airway irritants correlate with *in vivo* responses

**FIGURE 4:**
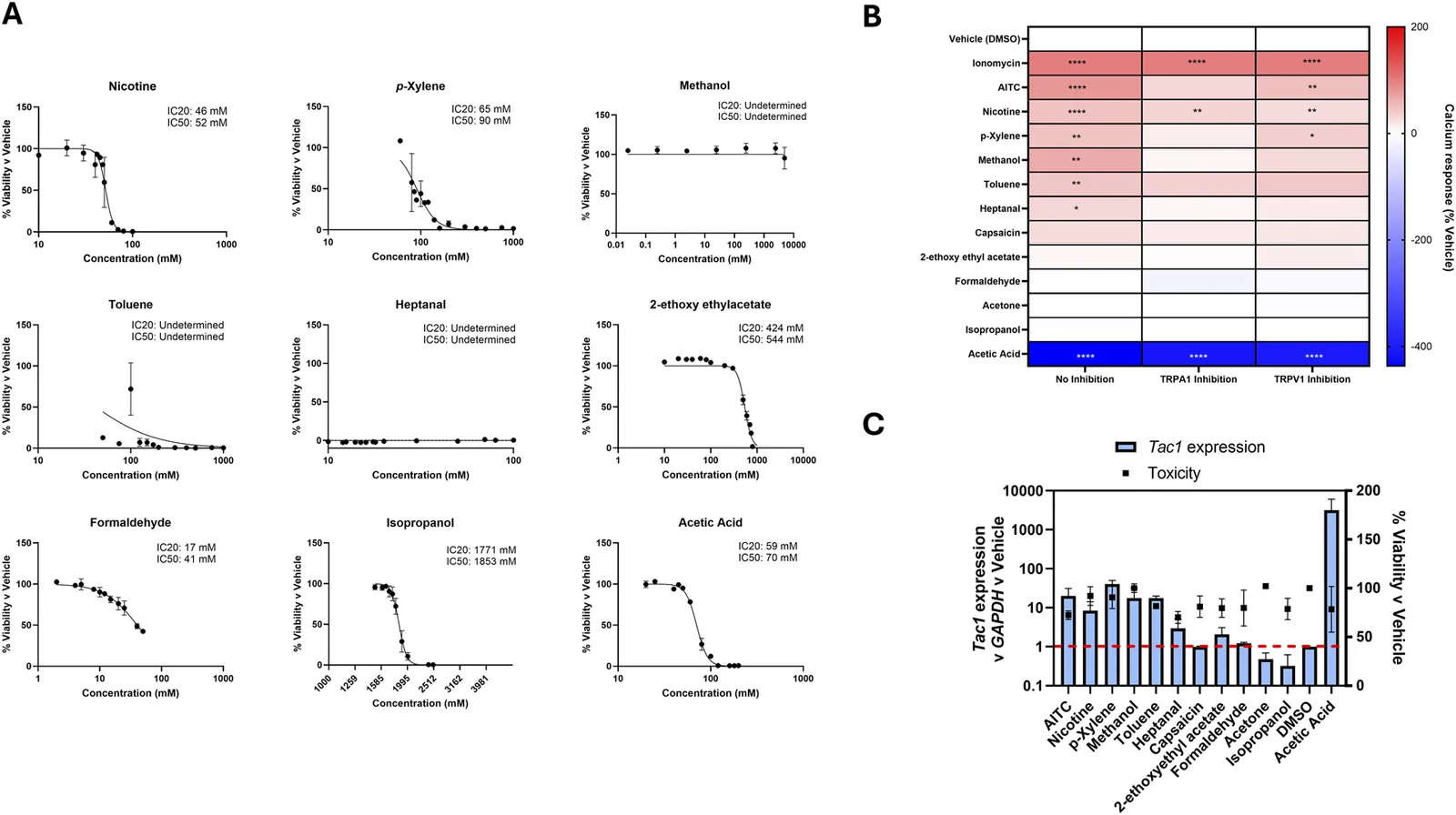
Further confirmation of toxicity, calcium response and *Tac1* expression by known airway irritants. A549 viability following a 24 h treatment with additional compounds was measured using WST-8 and IC_50_ and IC_20_ values calculated **(A)** and calcium influx measured following immediate application of each compound at a 1% (v/v) concentration in the presence of TRPA1 or TRPV1 inhibition **(B)**. Furthermore, *Tac1* expression for each of the compounds was investigated by qPCR following a 24 h treatment at a concentration that induced minimal toxicity (IC_20_), as confirmed by viability measured by RNA quantity extracted from cells **(C)**. Mean ± SEM (n = 3). One-way ANOVA with multiple comparisons. *p < 0.05, **p < 0.01, ***p < 0.001.

To further evaluate the ability of our *in vitro* model to identify airway irritants, we compared 24 h viability outcomes (Figure 4A) with calcium responses measured at a 1% (v/v) concentration to enable comparison between compounds when tested at the same concentrations (Figure 4B). Overall, viability and calcium signaling showed strong concordance. For example, nicotine, *p*-xylene, and heptanal each produced low IC_20_ values and elicited significant calcium responses under non-inhibited (TRPA1/TRPV1-active) conditions. AITC demonstrated a similar alignment between viability and calcium response (Figure 3A). In contrast, acetone (Figure 3C) and isopropanol (Figure 4A) - both associated with minimal airway irritation *in vivo* - showed low toxicity (IC_20_ values of 2,269 and 1771 mM, respectively) and did not induce calcium responses (Figure 4B).

Despite these general trends, certain compounds produced divergent outcomes. Methanol induced a calcium response without causing toxicity, whereas formaldehyde caused substantial toxicity but triggered a variable calcium response. These discrepancies underline the importance of integrating data from both assays when evaluating potential irritant risk.

To further leverage mechanistic pathways associated with airway irritation, *Tac1* expression was quantified following treatment with the full panel of test compounds (Figure 4C). In general, *Tac1* induction was greater for compounds that produced significant calcium responses. As previously demonstrated, the endogenous control gene, *GAPDH*, response remained consistent during compound treatments, with an average Ct value of 17.953 ± 0.578 SD observed.

Results from the *in vitro* viability, calcium response, and *Tac1* assays were then compared with historical *in vivo* irritancy data (Table 2). Acetone and isopropanol showed strong concordance with their high *in vivo* RD_50_ values (23,480 and 5,000 ppm, respectively), exhibiting low toxicity, negligible calcium responses, and minimal *Tac1* expression using our *in vitro* model. Several compounds known to cause pronounced irritation *in vivo* - including AITC, nicotine, *p*-xylene, and toluene - also produced high toxicity, robust calcium responses, and elevated *Tac1* expression in our model. As noted above, interpretation of compound risk requires consideration of all endpoints collectively. For instance, although formaldehyde induced a variable calcium response and did not induce *Tac1* expression, its high toxicity aligned with *in vivo* observations. Conversely, methanol elicited calcium and *Tac1* responses but only induced toxicity at high concentrations both *in vitro* and *in vivo*. Interestingly, methanol has previously been shown to increase intracellular calcium in mammalian cells, however, to our knowledge, its correlation with *Tac1* or substance P expression has not been investigated (Li et al., 1999). Furthermore, the discrepancy between *Tac1* expression and limited toxicity would require further investigation to highlight the mechanism involved.

**TABLE 2:** Comparison of *in vitro* viability, calcium response and *Tac1* expression with *in vivo* RD_50_ data.

Compound	Viability IC_20_ (mM)	Viability IC_50_ (mM)	Calcium response	Increased *Tac1* expression	*In vivo* RD_50_ (ppm)
AITC	0.0006	0.0028	Increased (*p < 0.0001*)	Yes	0.5–5
Nicotine	46	52	Increased (*p < 0.0001*)	Yes	5.28^†^
p-Xylene	65	90	Increased (*p < 0.01*)	Yes	1,325^†^
Methanol	>5,000	>5,000	Increased (*p < 0.01*)	Yes	25,222^†^
Toluene	<50	<50	Increased (*p < 0.01*)	Yes	3,373^†^
Heptanal	<10	<10	Increased (*p < 0.05*)	Yes	Not investigated
Capsaicin	0.273	>500	Increased (*ns*)	No	0.83^a^
2-Ethoxy ethyl acetate	424	544	No response	No	580^†^
Formaldehyde	17	41	No response	No	3.1^†^
Acetone	2,269	3,522	No response	No	23,480^†^
Isopropanol	1771	1853	No response	No	5,000^†^
Acetic acid	59	70	Decreased (*p < 0.0001*)	Yes	163^†^

^†^ Values retrieved from Schaper (1993), with additions from Nielsen et al. (2007), Dudek et al. (1992); Alarie et al. (1980). The range of values are retrieved from different experiments, including using different strains of mice.

^a^ Morris et al. (1985).

### 3.5 The organotypic, bronchial epithelial-derived model, EpiAirway™ demonstrates TRPA1-driven substance P responses

Compared with the lower airway, epithelial cells of the upper airway serve as an early defensive barrier, detecting inhaled environmental stimuli and shaping the body’s subsequent response. The bronchial-derived EpiAirway™ model offers several advantages over traditional submerged cell culture systems, including the ability to respond to compounds at physiologically relevant concentrations due to features such as ciliation and mucus production. Building on these attributes, we evaluated whether the EpiAirway™ model could also generate biomarkers associated with the onset of airway irritation, including TRPA1-mediated calcium signaling and substance P release. Integrating these mechanistic endpoints alongside phenotypically protective features of ciliation and mucus production, within a single model, may help to reduce false-positive classifications during compound risk assessment.

**FIGURE 5:**
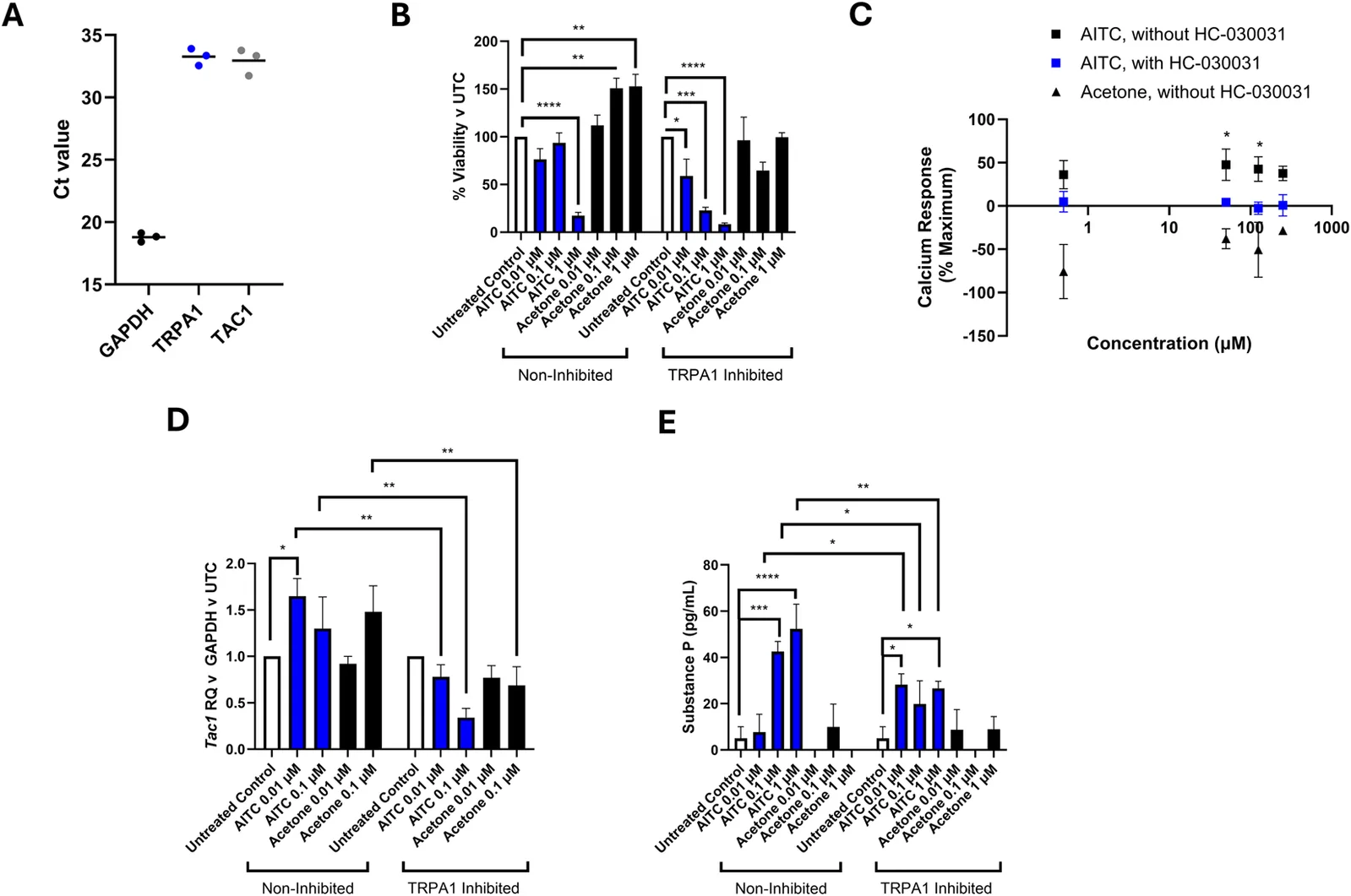
Development of assays for the measurement of airway irritation using the bronchial-derived EpiAirway™ model. Confirmation of presence of *TRPA1* and *Tac1* expression in EpiAirway™ tissues was measured by qPCR **(A)** followed by EpiAirway™ viability assessment by RNA yield following treatment with AITC and acetone in the absence and presence of TRPA1 inhibition **(B)**. Calcium influx was also measured in the absence and presence of TRPA1 inhibition **(C)**. Furthermore, changes in *Tac1* expression **(D)** and substance P release **(E)** were measured in EpiAirway™ tissues following treatment with a range of AITC or acetone concentrations in the absence and presence of TRPA1 inhibition. It should be noted that due to high toxicity measured following treatment with 1 μM AITC, gene expression could not be measured at this concentration. As a result, gene expression was not measured in tissues treated with 1 μM acetone for comparison purposes. Mean ± SEM (n = 3). One-way ANOVA with multiple comparisons. *p < 0.05, **p < 0.01, ***p < 0.001, ****p < 0.0001.

To determine the suitability of the EpiAirway™ model for producing irritation-related biomarkers, we first confirmed the presence of TRPA1 and *Tac1* transcripts, with Ct values of 33.271 (±0.67) and 32.954 (±1.08), respectively (Figure 5A). Tissues were then exposed for 24 h to a concentration range of AITC or acetone, with or without 1 μM HC-030031, and viability was assessed by RNA yield (Figure 5B). AITC produced a significant calcium response (p < 0.05), which was inhibited following treatment with 100 μM HC-030031 for 30 min (Figure 5C). To further confirm TRPA1-dependent substance P signaling, *Tac1* expression was measured following AITC or acetone treatment. A significant increase in *Tac1* expression was observed at 0.01 μM AITC treatment (*p* < 0.05), followed by a decrease at 0.1 μM AITC, likely reflecting high cytotoxicity at this concentration. When 0.01 μM AITC was tested in the presence of TRPA1 inhibition, *Tac1* expression was significantly reduced compared with non-inhibited conditions (*p* < 0.01), despite similar viability observed (∼50%), confirming TRPA1 involvement (Figure 5D).

Finally, to verify that *Tac1* transcription translated into substance P protein release, substance P levels were quantified by ELISA (Figure 5E). A concentration-dependent increase in substance P release was observed following AITC exposure, with significant elevations at 0.1 μM (*p* < 0.001) and 1 μM (*p* < 0.0001). TRPA1 inhibition significantly reduced substance P release at both concentrations (*p* < 0.05 and *p* < 0.01, respectively), further supporting TRPA1-driven substance P signaling in this model.

## 4 Discussion

Respiratory exposure to environmental irritants contributes to a range of acute and chronic airway disorders, and the prevalence of these conditions continues to increase despite the implementation of occupational exposure limits (OELs). Sensory irritation from inhaled chemicals is still assessed primarily using non-human animal models, most notably the RD_50_ assay (Alarie, 1973). Although RD_50_ values are informative for setting exposure thresholds and correlate well with lowest observed adverse effect levels (LOAELs) in humans (Kuwabara et al., 2007), a major limitation of this model is the substantial interspecies variability in ion channels, particularly TRPA1, that are central to chemosensory detection.

This variability in ion channel homology between species can lead to striking differences in channel pharmacology. For example, the human TRPA1 antagonists AMG5445 and AMG9090 act as agonists at the rat TRPA1 ortholog (Klionsky et al., 2007). In many cases, these species-specific differences arise from only a small number of amino acid substitutions. CMP1, which activates rat TRPA1 but inhibits human TRPA1, is functionally distinguished by Ala-946 and Met-949 in rat versus Ser-943 and Ile-946 in human (Chen et al., 2008). Similarly, the bimodal effects of menthol on mouse TRPA1, contrasted with its activation of human TRPA1, are determined by two residues in the pore region, Ser-876 and Thr-877 (Xiao et al., 2008). Even a single amino acid change can be decisive: substitution of Met268Pro in mouse TRPA1 converts caffeine from an agonist to an antagonist, and human TRPA1, activated by caffeine, naturally contains a proline at this position (Nagatomo et al., 2010).

Collectively, these examples illustrate that despite high overall conservation, subtle sequence differences between species can profoundly alter ion channel kinetics. Consequently, a compound’s ability to initiate downstream protective signaling pathways, such as the release of the neuro-inflammatory peptide substance P, may differ markedly between humans and commonly used experimental animal models, highlighting the need for species-relevant approaches to evaluating airway irritants.

Although substance P is classically released from neuronal populations to initiate inflammatory signaling, in this study we examined whether the human alveolar epithelial cell line A549 is capable of endogenous TRPA1–substance P signaling. TRPA1 expression has previously been reported in A549 cells, and its activation has been linked to cellular responses to wood smoke particulate matter; however, downstream biological consequences of TRPA1 activation in this context were not characterized (Shapiro et al., 2013). Additional studies in other cell types including rat dorsal root ganglia and human osteoarthritic chondrocytes have demonstrated that TRPA1 activation can drive inflammatory mediator release, including substance P, IL-6, and IL-1β (Nakamura et al., 2012; Nummenmaa et al., 2020; Lee et al., 2016; Nummenmaa et al., 2016). Moreover, the substance P receptor neurokinin-1 has been detected in A549 cells (Nguyen et al., 2023). Together, these independent findings suggest that A549 cells possess the molecular components necessary to support TRPA1-dependent neuro-inflammatory signaling. However, to our knowledge, endogenous TRPA1-driven substance P release has not been investigated.

To validate this potential, we first confirmed functional expression of both TRPA1 and TRPV1 in A549 cells using a flow cytometry - based calcium response assay (Figure 1). Although this approach has been applied previously in human B lymphocytes and macrophages, we sought to establish its suitability for calcium imaging in A549 cells (Bajnok et al., 2023; Schlautmann and Burgdorf, 2026). We therefore assessed calcium responses following exposure to a panel of chemicals known to elicit varying degrees of airway irritation in animal models (Figure 2A). Baseline fluorescence and cell viability, assessed using propidium iodide staining, remained stable across treatments, supporting the robustness of the assay for this application (Figure 2B). As expected, known irritants, including nicotine, *p*-xylene, and toluene, elicited clear calcium responses, whereas isopropanol, a compound associated with minimal irritation in humans, did not induce detectable calcium flux. These results indicate that the assay can discriminate between chemicals with differing irritancy profiles.

We next assessed concordance between calcium response and *Tac1* gene induction - a necessary precursor for substance P production, following TRPV1 and TRPA1 activation (Figure 3). Results indicated that *Tac1* expression was driven to a greater extent by TRPA1 activation, with TRPV1 activation inducing negligible *Tac1* expression, although a small (∼1.5-fold) increase was observed when capsaicin was added at 500 μM. Treatment with acetone did not induce *Tac1* expression. Furthermore, TRPA1-driven *Tac1* gene expression translated to substance P protein expression as measured by immunocytochemistry. In contrast to our results, TRPV1 activation has been demonstrated to induce substance P release in stomach and pancreatic sensory neurons (Gazzieri et al., 2007; Wick et al., 2006). However, it should be noted that in corneal sensory neurons, TRPV1 activation has been shown to facilitate, rather than directly induce, substance P release in TRPM8+ neurons (Li et al., 2019). As a result, further investigative work would be required to confirm the presence and effect of additional molecular markers that may be required for TRPV1 driven substance P release in A549 cells. Furthermore, it is possible that our model would demonstrate TRPV1 mediated substance P release when tested at higher concentrations. In our model, when testing TRPV1-mediated responses, although a concentration-related calcium response was observed on immediate application of capsaicin at the selected concentrations, the concentrations selected for 24 h treatments prior to viability and *Tac1* expression measurements, were not sufficient to induce an IC_50_ viability response. Therefore, greater concentrations of capsaicin may have induced higher levels of *Tac1* expression. Alternatively, TRPV1 may not have been localized to the plasma membrane, resulting in higher concentrations of capsaicin being required to evoke a calcium response than commonly used in neuronal cells (Koplas et al., 1997). Furthermore, this may explain the limited substance P expression observed following capsaicin treatment.

We next used the validated approach to test viability, calcium response and *Tac1* expression, following treatment with a panel of compounds known to induce varying levels of respiratory irritation using *in vivo* models (Figure 4). Overall, we observed strong concordance among viability, calcium responses, and *Tac1* expression, with a few notable exceptions. For instance, AITC, nicotine, *p*-xylene, and toluene all produced high toxicity, robust calcium influx, and elevated *Tac1* expression, aligning well with *in vivo* irritancy data. Methanol, however, induced a calcium response and *Tac1* expression without causing toxicity, underlining the importance of evaluating multiple biological endpoints when assessing irritant potential. Acetic acid further illustrated this point: despite an unusual concentration-dependent decrease in calcium response, it produced substantial toxicity and strong *Tac1* induction, consistent with its known irritant properties. This again highlights the need to interpret all mechanistic readouts collectively rather than relying on any single measure.

In contrast to the positive responses observed with known irritants, acetone and isopropanol - both associated with low irritancy *in vivo* (23,480 and 5,000 ppm, respectively), did not induce toxicity, calcium influx, or *Tac1* expression in our system. Beyond aligning well with established *in vivo* data (Table 2), the model also maintained stable baseline fluorescence and cell viability measurements throughout testing, as previously mentioned, confirming that the assay conditions were robust and suitable for reliably assessing calcium responses over the required duration.

The alveolar epithelium serves as an important barrier in the human lower airway; however, the nasal and bronchial epithelium constitute the primary interface with inhaled irritants. Therefore, we extended our findings from the alveolar A549 model to evaluate whether the bronchial-derived EpiAirway™ tissue exhibits comparable responses. The presence of structural features such as ciliation and mucus production enables the EpiAirway™ model to more closely recapitulate human airway physiology, making it a preferred platform for toxicity testing relative to monolayer systems like A549 cells. Despite this advantage, the expression of TRPA1 and Substance P within the EpiAirway™ model has not, to our knowledge, been previously characterized.

In this study, we first confirmed the presence of both TRPA1 and Substance P in EpiAirway™ tissues. We then demonstrated that TRPA1 activation elicited robust calcium flux and induced substance P expression, mirroring the responses observed in our A549-based system. These findings highlight the potential of the EpiAirway™ model as a physiologically relevant screening platform for identifying airway irritants (Figure 5). Despite these findings, we unexpectedly found that the presence of HC-030031 resulted in higher levels of toxicity in the EpiAirway™ model. We suggest that this may be a result of loss of protective mechanisms provided by TRPA1 activation, such as responses linked to oxidative stress and inflammatory mediators. Indeed, the presence of HC-030031 also reduced the secretion of substance P, which has demonstrated protective mechanisms in neuronal cells. Furthermore, substance P has demonstrated protective mechanisms in lung cells following exposure to cigarette smoke and respiratory viruses (Ahn et al., 2023; Hasenöhrl et al., 2000; Harris and Witten, 2003; Mehboob et al., 2024). Furthermore, the EpiAirway™ model demonstrated higher levels of toxicity when treated with AITC only, compared to the A549 cells. We suggest that this may result from the lower overall expression of substance P, as measured by *Tac1* expression, observed in the EpiAirway™ model, however this requires further investigation, for example, measurement of A549 substance P release by ELISA, as was performed for the EpiAirway™ model, may provide additional insights.

In conclusion, we have verified that A549 cells and the EpiAirway™ model express paramount molecular biomarkers implicated in sensory irritation of the human airway (Martinez and Eling, 2019). Moreover, to our knowledge, this study provides the first evidence that these models can autonomously regulate Substance P production following activation of endogenously expressed TRPA1. We subsequently leveraged this capability to establish an innovative *in vitro* assay capable of screening compounds for their modulation of these molecular biomarkers. This assay could also be utilized alongside other *in vitro* models used in respiratory hazard assessment, such as those that focus on respiratory sensitization, for example, ALIsens® and GARD® air (Gutleb et al., 2026; Forreryd et al., 2015). Despite these findings, future work should aim to expand the number of compounds tested using this model to further refine its potential as a screening tool for respiratory irritation risk. These additional data would help to provide criteria by which compounds can be categorized based on their irritation potential. Furthermore, our model does not account for compound effects in the air phase. The immediate calcium responses induced on ion channel activation makes their measurement during concurrent compound exposure at the air liquid interface difficult. Despite this, future studies may investigate downstream signaling responses, such as substance P release, following exposure to compounds in their gaseous forms (Nielsen and Wolkoff, 2017).

## Data Availability

The original contributions presented in the study are included in the article/supplementary material; further inquiries can be directed to the corresponding authors.

## References

[B1] Ahn W. Chi G. Kim S. Son Y. Zhang M. (2023). Substance P reduces infarct size and mortality after ischemic stroke, possibly through the M2 polarization of microglia/macrophages and neuroprotection in the ischemic rat brain. Cell Mol. Neurobiol. 43 (5), 2035–2052. 10.1007/s10571-022-01284-7 36112332 PMC11412183

[B2] Alarie Y. (1973). Sensory irritation by airborne chemicals. CRC Crit. Rev. Toxicol. 2 (3), 299–363. 10.3109/10408447309082020 4131690

[B3] Alarie Y. (1981). Bioassay for evaluating the potency of airborne sensory irritants and predicting acceptable levels of exposure in man. Food Cosmet. Toxicol. 19 (5), 623–626. 10.1016/0015-6264(81)90513-7 7308905

[B4] Alarie Y. Kane L. Barrow C. (1980). “Sensory irritation: the use of an animal model to establish acceptable exposure to airborne chemical irritants,” in Toxicology: Principles and Practice. Editor Reeves A. L. (John Wiley and Sons), 48–92.

[B5] Bajnok A. Serény-Litvai T. Temesfői V. Nörenberg J. Herczeg R. Kaposi A. (2023). An optimized flow cytometric method to demonstrate the differentiation stage-dependent Ca^2+^ flux responses of peripheral human B cells. Int. J. Mol. Sci. 24 (10), 9107. 10.3390/ijms24109107 37240453 PMC10219102

[B6] Baker A. J. Brandes R. Schreur J. H. Camacho S. A. Weiner M. W. (1994). Protein and acidosis alter calcium-binding and fluorescence spectra of the calcium indicator indo-1. Biophys. J. 67 (4), 1646–1654. 10.1016/S0006-3495(94)80637-X 7819496 PMC1225526

[B7] Baur X. Bakehe P. Vellguth H. (2012). Bronchial asthma and COPD due to irritants in the workplace - an evidence-based approach. J. Occup. Med. Toxicol. 7 (1), 19. 10.1186/1745-6673-7-19 23013890 PMC3508803

[B8] Bautista D. M. Pellegrino M. Tsunozaki M. (2013). TRPA1: a gatekeeper for inflammation. Annu. Rev. Physiol. 75, 181–200. 10.1146/annurev-physiol-030212-183811 23020579 PMC4041114

[B9] Bessac B. F. Jordt S. E. (2008). Breathtaking TRP channels: TRPA1 and TRPV1 in airway chemosensation and reflex control. Physiol. (Bethesda) 23, 360–370. 10.1152/physiol.00026.2008 19074743 PMC2735846

[B10] Bos P. M. Busschers M. Arts J. H. (2002). Evaluation of the sensory irritation test (alarie test) for the assessment of respiratory tract irritation. J. Occup. Environ. Med. 44 (10), 968–976. 10.1097/00043764-200210000-00017 12391777

[B11] Chen J. Zhang X. F. Kort M. E. Huth J. R. Sun C. Miesbauer L. J. (2008). Molecular determinants of species-specific activation or blockade of TRPA1 channels. J. Neurosci. 28 (19), 5063–5071. 10.1523/JNEUROSCI.0047-08.2008 18463259 PMC6670736

[B12] Dalton P. (2003). Upper airway irritation, odor perception and health risk due to airborne chemicals. Toxicol. Lett. 140-141, 239–248. 10.1016/s0378-4274(02)00510-6 12676471

[B13] Dudek B. R. Short R. D. Brown M. A. Roloff M. V. (1992). Structure-activity relationship of a series of sensory irritants. J. Toxicol. Environ. Health 37 (4), 511–518. 10.1080/15287399209531689 1464906

[B14] Forreryd A. Johansson H. Albrekt A. S. Borrebaeck C. A. Lindstedt M. (2015). Prediction of chemical respiratory sensitizers using GARD, a novel *in vitro* assay based on a genomic biomarker signature. PLoS One 10 (3), e0118808. 10.1371/journal.pone.0118808 25760038 PMC4356558

[B15] Gazzieri D. Trevisani M. Springer J. Harrison S. Cottrell G. S. Andre E. (2007). Substance P released by TRPV1-expressing neurons produces reactive oxygen species that mediate ethanol-induced gastric injury. Free Radic. Biol. Med. 43 (4), 581–589. 10.1016/j.freeradbiomed.2007.05.018 17640568

[B16] Gerhold K. A. Bautista D. M. (2008). TRPA1: irritant detector of the airways. J. Physiol. 586 (14), 3303. 10.1113/jphysiol.2008.158030 18625802 PMC2538811

[B17] Gutleb A. C. Blumbach K. Burla S. Faulhammer F. Sommer T. M. Wiench K. (2026). A Novel *in vitro* Alveolar Model (ALIsens®) for Hazard Assessment of Methyl Methacrylate: No Evidence for Respiratory Sensitisation Potential. J NAM. 10.1016/j.namjnl.2025.100070

[B18] Harris D. T. Witten M. (2003). Aerosolized substance P protects against cigarette-induced lung damage and tumor development. Cell Mol. Biol. (Noisy-le-grand) 49 (2), 151–157. 12887098

[B19] Hasenöhrl R. U. Souza-Silva M. A. Nikolaus S. Tomaz C. Brandao M. L. Schwarting R. K. (2000). Substance P and its role in neural mechanisms governing learning, anxiety and functional recovery. Neuropeptides 34 (5), 272–280. 10.1054/npep.2000.0824 11049731

[B20] Jung Y. S. Chiang S. Athni T. S. Shah J. McCurdy K. Yu S. E. (2025). Characterizing everyday exposure to volatile organic compounds and upper respiratory health effects. Sci. Rep. 15 (1), 23555. 10.1038/s41598-024-81873-2 40603863 PMC12222567

[B21] Kim D. Choi J. (2026). Application of *in vitro* new approach methodologies data to chemical risk assessment: current status and perspectives toward next generation risk assessment. Front. Toxicol. 8, 1754231. 10.3389/ftox.2026.1754231 41923925 PMC13038229

[B22] Klionsky L. Tamir R. Gao B. Wang W. Immke D. C. Nishimura N. (2007). Species-specific pharmacology of trichloro(sulfanyl)ethyl benzamides as transient receptor potential ankyrin 1 (TRPA1) antagonists. Mol. Pain 3, 39. 10.1186/1744-8069-3-39 18086308 PMC2222611

[B23] Koplas P. A. Rosenberg R. L. Oxford G. S. (1997). The role of calcium in the desensitization of capsaicin responses in rat dorsal root ganglion neurons. J. Neurosci. 17 (10), 3525–3537. 10.1523/JNEUROSCI.17-10-03525.1997 9133377 PMC6573672

[B57] Kuwabara Y. Alexeeff G. V. Broadwin R. Salmon A. G. (2007). Evaluation and application of the RD50 for determining acceptable exposure levels of airborne sensory irritants for the general public. Environ. Health Perspect. 115 (11), 1609–1616. 10.1289/ehp.9848 18007993 PMC2072859

[B24] Lee K. I. Lee H. T. Lin H. C. Tsay H. J. Tsai F. C. Shyue S. K. (2016). Role of transient receptor potential ankyrin 1 channels in alzheimer's disease. J. Neuroinflammation 13 (1), 92. 10.1186/s12974-016-0557-z 27121378 PMC4847235

[B25] Lee M. Hwang J. H. Lim K. M. (2017). Alternatives to *in vivo* draize rabbit eye and skin irritation tests with a focus on 3D reconstructed human cornea-like epithelium and epidermis models. Toxicol. Res. 33 (3), 191–203. 10.5487/TR.2017.33.3.191 28744350 PMC5523559

[B26] Li W. Zheng T. Wang J. Altura B. T. Altura B. M. (1999). Methanol elevates cytosolic calcium ions in cultured canine cerebral vascular smooth muscle cells: possible relation to CNS toxicity. Alcohol 18 (2-3), 221–224. 10.1016/s0741-8329(99)00007-5 10456574

[B27] Li F. Yang W. Jiang H. Guo C. Huang A. J. W. Hu H. (2019). TRPV1 activity and substance P release are required for corneal cold nociception. Nat. Commun. 10 (1), 5678. 10.1038/s41467-019-13536-0 31831729 PMC6908618

[B28] Macpherson L. J. Xiao B. Kwan K. Y. Petrus M. J. Dubin A. E. Hwang S. (2007). An ion channel essential for sensing chemical damage. J. Neurosci. 27 (42), 11412–11415. 10.1523/JNEUROSCI.3600-07.2007 17942735 PMC6673017

[B29] Martinez J. M. Eling T. E. (2019). Activation of TRPA1 by volatile organic chemicals leading to sensory irritation. ALTEX 36 (4), 572–582. 10.14573/altex.1811012 31026039 PMC6829608

[B30] Maruta H. Yamashita H. (2020). Acetic acid stimulates G-protein-coupled receptor GPR43 and induces intracellular calcium influx in L6 myotube cells. PLoS One 15 (9), e0239428. 10.1371/journal.pone.0239428 32997697 PMC7526932

[B31] Mehboob R. Oehme P. Anwar T. von Kries J. P. (2024). Substance P - a regulatory peptide with defense and repair functions. Results and perspectives for the fight against COVID-19. Front. Neurol. 15, 1370454. 10.3389/fneur.2024.1370454 38872816 PMC11169637

[B32] Morris J. B. Symanowicz P. T. Olsen J. E. Thrall R. S. Cloutier M. M. Hubbard A. K. (1985). Immediate sensory nerve-mediated respiratory responses to irritants in healthy and allergic airway-diseased mice. J. Appl. Physiol. 94 (4), 1563–1571. 10.1152/japplphysiol.00572.2002 12626476

[B33] Mucchietto V. Fasoli F. Pucci S. Moretti M. Benfante R. Maroli A. (2018). α9-and α7-containing receptors mediate the pro-proliferative effects of nicotine in the A549 adenocarcinoma cell line. Br. J. Pharmacol. 175 (11), 1957–1972. 10.1111/bph.13954 28726253 PMC5980168

[B34] Müller I. Alt P. Rajan S. Schaller L. Geiger F. Dietrich A. (2022). Transient receptor potential (TRP) channels in airway toxicity and disease: an update. Cells 11 (18), 2907. 10.3390/cells11182907 36139480 PMC9497104

[B35] Nagatomo K. Ishii H. Yamamoto T. Nakajo K. Kubo Y. (2010). The Met268Pro mutation of mouse TRPA1 changes the effect of caffeine from activation to suppression. Biophys. J. 99 (11), 3609–3618. 10.1016/j.bpj.2010.10.014 21112285 PMC2998636

[B36] Nakamura Y. Une Y. Miyano K. Abe H. Hisaoka K. Morioka N. (2012). Activation of transient receptor potential ankyrin 1 evokes nociception through substance P release from primary sensory neurons. J. Neurochem. 120 (6), 1036–1047. 10.1111/j.1471-4159.2011.07628.x 22182301

[B37] Nguyen L. P. Cho M. Nguyen T. U. Park H. K. Nguyen H. T. Mykhailova K. (2023). Neurokinin-2 receptor negatively modulates substance P responses by forming complex with Neurokinin-1 receptor. Cell Biosci. 13 (1), 212. 10.1186/s13578-023-01165-6 37968728 PMC10652611

[B38] Nielsen G. D. Wolkoff P. (2017). Evaluation of airborne sensory irritants for setting exposure limits or guidelines: a systematic approach. Regul. Toxicol. Pharmacol. 90, 308–317. 10.1016/j.yrtph.2017.09.015 28911939

[B39] Nielsen G. D. Wolkoff P. Alarie Y. (2007). Sensory irritation: risk assessment approaches. Regul. Toxicol. Pharmacol. 48 (1), 6–18. 10.1016/j.yrtph.2006.11.005 17241726

[B40] Norões M. M. Santos L. G. Gavioli E. C. de Paula Soares Rachetti V. Otuki M. F. de Almeida C. D. (2019). Role of TRPA1 receptors in skin inflammation induced by volatile chemical irritants in mice. Eur. J. Pharmacol. 858, 172460. 10.1016/j.ejphar.2019.172460 31228448

[B41] Nummenmaa E. Hämäläinen M. Moilanen L. J. Paukkeri E. L. Nieminen R. M. Moilanen T. (2016). Transient receptor potential ankyrin 1 (TRPA1) is functionally expressed in primary human osteoarthritic chondrocytes. Arthritis Res. Ther. 18 (1), 185. 10.1186/s13075-016-1080-4 27515912 PMC4982008

[B42] Nummenmaa E. Hämäläinen M. Pemmari A. Moilanen L. J. Tuure L. Nieminen R. M. (2020). Transient receptor potential ankyrin 1 (TRPA1) is involved in upregulating Interleukin-6 expression in osteoarthritic chondrocyte models. Int. J. Mol. Sci. 22 (1), 87. 10.3390/ijms22010087 33374841 PMC7794684

[B43] OECD (2015). Test No. 439: *In vitro* Skin Irritation: Reconstructed Human Epidermis Test Method. Paris: OECD Publishing.

[B44] OECD (2016). Test No. 431: *In vitro* Skin Corrosion: Reconstructed Human Epidermis (RHE) Test Method. Paris: OECD Publishing.

[B46] Rohrbach P. Friedrich O. Hentschel J. Plattner H. Fink R. H. Lanzer M. (2005). Quantitative calcium measurements in subcellular compartments of plasmodium falciparum-infected erythrocytes. J. Biol. Chem. 280 (30), 27960–27969. 10.1074/jbc.M500777200 15927958

[B47] Sándor K. Helyes Z. Elekes K. Szolcsányi J. (2009). Involvement of capsaicin-sensitive afferents and the transient receptor potential vanilloid 1 receptor in xylene-induced nocifensive behaviour and inflammation in the mouse. Neurosci. Lett. 451 (3), 204–207. 10.1016/j.neulet.2009.01.016 19159661

[B58] Schlautmann L. Burgdorf S. (2026). Protocol to assess calcium signaling in response to metabolic alterations by flow cytometry and live-cell imaging. STAR Protoc. 7 (2), 104435. 10.1016/j.xpro.2026 41838718 PMC12991838

[B48] Schaper M. (1993). Development of a database for sensory irritants and its use in establishing occupational exposure limits. Am. Ind. Hyg. Assoc. J. 54 (9), 488–544. 10.1080/15298669391355017 8379496

[B49] Shapiro D. Deering-Rice C. E. Romero E. G. Hughen R. W. Light A. R. Veranth J. M. (2013). Activation of transient receptor potential ankyrin-1 (TRPA1) in lung cells by wood smoke particulate material. Chem. Res. Toxicol. 26 (5), 750–758. 10.1021/tx400024h 23541125 PMC3670828

[B50] Song J. Kang J. Lin B. Li J. Zhu Y. Du J. (2017). Mediating role of TRPV1 ion channels in the Co-exposure to PM2.5 and formaldehyde of Balb/c mice asthma model. Sci. Rep. 7 (1), 11926. 10.1038/s41598-017-11833-6 28931832 PMC5607312

[B51] Springer J. Pleimes D. Scholz F. R. Fischer A. (2005). Substance P mediates AP-1 induction in A549 cells via reactive oxygen species. Regul. Pept. 124 (1-3), 99–103. 10.1016/j.regpep.2004.07.004 15544846

[B52] Sun H. J. Jia Y. F. Ma X. L. (2017). Alpha5 nicotinic acetylcholine receptor contributes to nicotine-induced lung cancer development and progression. Front. Pharmacol. 8, 573. 10.3389/fphar.2017.00573 28878681 PMC5572410

[B53] Wallbanks S. Griffiths B. Thomas M. Price O. J. Sylvester K. P. (2024). Impact of environmental air pollution on respiratory health and function. Physiol. Rep. 12 (16), e70006. 10.14814/phy2.70006 39175108 PMC11341277

[B54] Wick E. C. Hoge S. G. Grahn S. W. Kim E. Divino L. A. Grady E. F. (2006). Transient receptor potential vanilloid 1, calcitonin gene-related peptide, and substance P mediate nociception in acute pancreatitis. Am. J. Physiol. Gastrointest. Liver Physiol. 290 (5), G959–G969. 10.1152/ajpgi.00154.2005 16399878

[B55] Xiao B. Dubin A. E. Bursulaya B. Viswanath V. Jegla T. J. Patapoutian A. (2008). Identification of transmembrane domain 5 as a critical molecular determinant of menthol sensitivity in mammalian TRPA1 channels. J. Neurosci. 28 (39), 9640–9651. 10.1523/JNEUROSCI.2772-08.2008 18815250 PMC2678945

[B56] Xu Y. Huang C. Deng H. Jia J. Wu Y. Yang J. (2018). TRPA1 and substance P mediate stress induced duodenal lesions in water immersion restraint stress rat model. Turk J. Gastroenterol. 29 (6), 692–700. 10.5152/tjg.2018.17817 30381276 PMC6284685

